# The Future of Medicine

**Published:** 1918-09-07

**Authors:** 


### The Hospital

The Workers' Newspaper of Administrative Medicine and Institutional Life, Administration, National Insurance and Health.

No. 1683, Vol. LXIV. SATURDAY, SEPTEMBER 7, 1918 PRICE TWOPENCE

### THE FUTURE OF MEDICINE.

The future, even the immediate future, of the medical profession does not offer an untroubled prospect for those who at the present moment have to try to estimate its probable value and developments. Change, and perhaps considerable change, is in the air, and it is anticipated on all hands that much of the old order is about to pass away. What the new status and structure will be may well puzzle even an expert judgment. Still more may the outcome seem uncertain to the uninitiated layman. Yet, at this time of the year especially, many lay minds have to contemplate the problem. The medical schools are about to reopen their doors, and a medical career is presented as a possibility to adventurous youth faced by the necessity of making a choice. Both the youngsters and their advisers merit more than a word of sympathy. The decision, always important and often difficult, is just now complicated, so far as medicine is concerned, by prevailing uncertainties. In former and less agitated days the disciple, it is true, might hesitate over his own inclinations and desires. But at least he had before him a fairly definite offer. The possibilities were clean-cut and had been established by the habit of years and even of generations. To enter a medical school, to pass the necessary examinations, to commence practice, and thus to gain the confidence of more or less numerous patients and to attain a certain social status, formed a line of action which had been traced again and again, and the meaning of the choice was free from all ambiguity. The prospect invited or did not invite. It stood on its merits and had no shadowy outlines. But so much cannot be said to-day, and all who have to judge the future must find in the new possibilities an added burden of perplexity.

This sense of impending change was brought into being some years prior to the war, but the war has emphasised it and made it acute, so that at the present moment it has reached the position of a general conviction and anticipation. The influences responsible for such a position arrive from two different sources. On the one hand is a keener appreciation of the national value of health in the community. On the other is the development of more exact and scientific inquiries in the methods of medical practice. If the nation is to be efficient, the health capacity and fitness capacity of its units must be preserved, and the average standard brought to a higher level. The medical measures necessary to secure this end must therefore be widely or universally available. Such, in a phrase, are the currents of opinion which move the political and social worlds to look to the medical profession for guidance and co-operation, and which stir the profession in its turn with the desire to contribute a larger measure of service to the cause of national welfare. Alike from one side and from the other it is recognised that these ambitions cannot be realised save by some scheme of medical organisation, and it is this conviction which provides the new point of departure.

The medical practitioner has hitherto found his main responsibility in his duties to his individual patients, and he has been largely free from other challenge. But obviously, if organisation is to take the place of independent judgment and action, both the atmosphere and the actualities of medical practice will undergo a change. In greater or less measure there will be authority and control or, at the very least, suggestion and pressure. A question of high importance is, By whom will these influences be exercised? And it is the existing uncertainty of the response which necessarily introduces a sense of obscurity in the minds of those who have to judge whether it is wise or unwise to elect for medical discipleship. If the new arrangements, whatever they be, are to be imposed on the profession from without, and are to include the rules and regulations and penalties which are so dear to the mind of congenital and acquired bureaucracy, medical practice may have a scheme and order which it does not at present possess. But it will hardly be an attractive career to such as seek opportunities for independence and initiative and who are little concerned with either the frowns or the smiles of those set in high places. The position will doubtless be a different one if the profession shows a readiness and ability to meet from within the new demands for co-operation and co-ordination. In such an event the aim will be to multiply for the practitioner opportunities for usefulness rather than to impose on him the inspired commands of constituted authorities. The issue between these two principles

**The Future of Medicine** *(continued)*.

is still in debate, and the future of the medical profession and the quality of those attracted to its ranks will depend very largely upon the outcome of the struggle.

Yet with all the uncertainties of the future we readily find a word of welcome and encouragement for the new recruits. They can be but little disturbed by the considerations which agitate the established practitioner. For them, medicine, according to a well-established tradition, offers a career worthy of generous minds and resolute hearts, and we do not hesitate to commend it as a discipline of high value for those who practise it and rich in opportunities for work and service and achievement. If there are few gauds in the prospect there are great realities, and it is the realities which count and abide. The future has indeed its uncertainties, but for our part we are not able to believe that the freedom of thought and speech and action which has hitherto characterised British medicine can be supplanted by the autocratic decrees either of well-drilled bureaucrats or of selected superior persons.

